# Prevalence of dementia in Singapore: Changes across a decade

**DOI:** 10.1002/alz.14485

**Published:** 2025-01-27

**Authors:** Mythily Subramaniam, Edimansyah Abdin, PV Asharani, Kumarasan Roystonn, Fiona Devi, Wang Peizhi, Saleha Shafie, Vathsala Sagayadevan, Anitha Jeyagurunathan, Boon Yiang Chua, Bernard Tan, Janhavi Ajit Vaingankar, Fengyuan Yao, Harish Magadi, Stefan Ma, Wai Leng Chow, Paul McCrone, Derrick Heng, Rathi Mahendran, Martin Prince, Li Ling Ng, Siow Ann Chong

**Affiliations:** ^1^ Research Division, Institute of Mental Health Singapore Singapore; ^2^ Saw Swee Hock School of Public Health National University of Singapore Singapore Singapore; ^3^ Lee Kong Chian School of Medicine Singapore Singapore; ^4^ Department of Geriatric Psychiatry Institute of Mental Health Singapore Singapore; ^5^ Ministry of Health Singapore Singapore; ^6^ School of Health Sciences University of Greenwich Greenwich London UK; ^7^ Department of Psychological Medicine National University of Singapore Singapore Singapore; ^8^ Kings College London Strand London UK; ^9^ Changi General Hospital Singapore Singapore

**Keywords:** 10/66 protocol, Asian, dementia, epidemiology, multi‐ethnic

## Abstract

**INTRODUCTION:**

The study aimed to assess changes in the prevalence of dementia in Singapore over the past decade.

**METHODS:**

The Well‐being of the Singapore Elderly (WiSE) 2023 and the WiSE 2013 studies were comprehensive, single‐phase, cross‐sectional surveys conducted among older adults aged ≥60 years in Singapore. WiSE 2023 included 2010 older adults and 1798 informants, whereas WiSE 2013 comprised 2565 older adults and 2421 informants.

**RESULTS:**

The weighted prevalence of dementia based on the 10/66 Diagnostic Research Group (DRG) criteria was 8.8% (95% confidence interval [CI]: 7.3–10.7) in the WiSE 2023 study compared to 10% in the WiSE 2013 study (95% CI: 8.7–11.5). The proportion of those with undiagnosed dementia decreased significantly from 70.6% in WiSE 2013 to 51.5% in WiSE 2023 (*p*‐value = 0.002).

**DISCUSSION:**

A non‐significant 12% reduction (95% CI: 1.1–3.5) in dementia prevalence was observed in Singapore over a decade, based on the WiSE 2013 and WiSE 2023 studies.

**Highlights:**

- The prevalence of dementia decreased from 10% to 8.8% over a decade in Singapore.
- The prevalence varied by age group, ethnicity, employment status, and health factors.
- The prevalence of undiagnosed dementia decreased significantly from 70.6% to 51.5%.

## 1 BACKGROUND

Dementia is a highly prevalent neurodegenerative disorder, with the World Health Organization (WHO) identifying it as a global public health priority.^1^ The syndrome is characterized by cognitive difficulties that progressively affect a person's ability to function independently.

The prevalence of dementia varies significantly across countries. Methodological differences, including the criteria for dementia diagnosis, the cognitive assessment tools used, and the type of sample used, that is, administrative data, registries, population surveys, and so on,^2^ limit comparisons across countries. However, several common risk factors of dementia have been identified across studies, which include less education, hearing impairment, vision loss, smoking, obesity, physical inactivity, diabetes mellitus (DM), high cholesterol, hypertension, depression, low social contact, excessive alcohol consumption, traumatic brain injury, and air pollution.^3^

**RESEARCH IN CONTEXT**

1. **Systematic review**: We reviewed the relevant literature using PubMed. Changes in the prevalence of dementia across time were inconsistent across countries. Although in some countries the estimates of the prevalence of dementia have likely decreased or remained stable over time, in others, they have increased. Thus, although studies in the United States and the United Kingdom found a decrease in the prevalence of dementia over time, those from Japan saw an increase in the prevalence of dementia.
2. **Interpretation**: A second nationwide study of older adults was conducted in Singapore to establish and compare the changes in the prevalence of dementia. The research team put stringent quality control measures in place to ensure the validity and reliability of the data collected. The results indicated that the prevalence of dementia using the 10/66 criteria was 8.8% in the older adult population. Translating this figure into Singapore's population in 2022, would mean that 73,918 older adults had dementia. Comparing the prevalence rate of dementia in Well‐being of the Singapore Elderly (WiSE) 2023 to that of 10% observed in WiSE 2013, a decrease of 1.2% percentage points or 12% was observed. However, the reduction in the prevalence of dementia between the two surveys was not statistically significant.
3. **Future directions**: Although the decrease in the prevalence of dementia is encouraging, active aging and dementia awareness initiatives, as well as strengthening primary care to ensure early diagnosis and treatment of those with dementia, must continue to be well‐funded. These programs must be evaluated to ensure that they continue to impact Singaporeans positively and that the gains observed are maintained.

Trends in dementia prevalence over time have been mixed. Studies suggest a decline in some countries (e.g., USA and UK), whereas in others, the prevalence has remained stable (Sweden) or has increased over time (e.g., Japan).^4^ Such differences emphasize the variances in the risk and protective factors across countries and the need for population‐specific studies to gain a deeper understanding of the mechanisms underpinning the changes and the impact of policy‐driven programs and interventions targeted at reducing the prevalence of dementia.

Singapore is a Southeast Asian city‐state comprising a multi‐ethnic population of Chinese, Malay, Indian, and other ethnicities. The first in‐depth study on dementia prevalence, the Well‐being of the Singapore Elderly (WiSE) study, completed in 2013, found that 10% of Singaporeans 60 years of age and older met the criteria for 10/66 dementia.^5^ Older age, lower education level, homemaker or retired status, and a history of stroke were associated with a higher likelihood of 10/66 dementia. The term 10/66 coined by the 10/66 DRG referred to the 66% of people with dementia who were living in developing countries and the less than 10% of population‐based research carried out in those settings. The 10/66 criteria for dementia is defined as those scoring above a cutpoint of predicted probability of the Diagnostic and Statistical Manual of Mental Disorders (Fourth Edition) (DSM‐IV) dementia from the logistic regression equation developed in the 10/66 international pilot study.^6^

The results of the WiSE 2013 study led to a further expansion and strengthening of Singapore's approach to dementia awareness, care, and prevention, to counter the growing challenges posed by a rapidly aging population.^7^ The Singapore government announced a $3 billion Action Plan for Successful Ageing in 2015 to create opportunities for seniors to learn, volunteer, and live independently.^8^ Under the Community Mental Health Masterplan launched in 2017, the Community Outreach Team also known as Community Resource, Engagement and Support Team (CREST) program worked with older adults to promote early recognition of at‐risk individuals, provide emotional support, and link individuals and their caregivers to relevant health and social care services when necessary. In addition, memory clinics were established in primary care clinics to improve access to dementia diagnosis and care management in the community.^9^

The need to monitor the trends in the prevalence of dementia as populations age with increasing life expectancy is paramount for health care planning and resource allocation. On the one hand, the prevalence of dementia may have been reduced (by lowering incidence) over time by the various programs initiated in Singapore. On the other, the impact of the coronavirus disease 2019 (COVID‐19) infection on older adults’ cognition,^10^ reduced exercise, as well as increased rates of depression and loneliness may have led to a worsening of cognitive decline among older adults during the pandemic.^11^ The establishment of memory clinics and greater awareness of dementia in Singapore has led to early help‐seeking and the increased identification of mild cases,^9^ which could potentially increase the prevalence of dementia. However, more adults with subjective cognitive decline and diagnosis of cognitive impairment/no dementia are also seeking help in memory clinics. This enhanced health care access could result in better management of comorbid conditions, health education, and referrals to activity centers, potentially preventing or delaying the onset of dementia.

It is also in line with the recommendations that all countries should monitor the trends of dementia prevalence and disease burden through nationally representative surveys undertaken over time.^12^ Hence, in 2022 the second WiSE study (WiSE 2023) was launched with the primary aims of (1) establishing the prevalence of dementia in the older adult population (60 years of age or older) of Singapore residents, (2) tracking the changes in the prevalence of dementia since the last study conducted a decade ago, (3) identifying factors associated with dementia, and (4) examining the treatment gap of dementia.

## 2 METHODS

The WiSE 2023 study was a comprehensive single‐phase, cross‐sectional survey that was conducted using the same methodology as the first WiSE (WiSE 2013) study.^5^ The sampling frame (administrative database used), sampling methodology, and invitation letter format were similar across the two studies and are described in detail in the following sections.

### 2.1 Sampling strategy

From an administrative database that comprises the names, ages, and addresses of Singapore citizens and permanent residents, older adults 60 years of age or older residing in Singapore, including those living in nursing homes, were randomly selected using a disproportionate stratified sampling method. In this sampling method, participants were stratified according to three age groups (60 to 74; 75 to 84, and 85 and older) and four ethnic groups (Chinese, Malay, Indian, and Others) to create 12 sampling strata. We then randomly sampled individuals within each stratum where older age and minority ethnic groups were oversampled to ensure sufficient sample sizes and precision for subgroup analysis. We oversampled (1) individuals 75 years of age and older to account for the potential low response in this age group due to family members being less likely to provide access to them given their advanced age and frailty; more of them being in long‐term facilities, which also leads to reluctance on the part of family members to provide contact details and access as well as the higher likelihood of mortality in this age group (the oversampling ensured that despite the risk of poor response in this group, we would have adequate data size for statistical power to detect statistical differences and also for further data analysis, if needed, among this group); and (2) those belonging to Indian and Malay ethnicities to ensure sufficient sample size in the two minority ethnic groups. Hence, sampling weights, which are inversely proportional to the respondents’ probability of selection within the stratum, were generated and incorporated during the analysis to account for the disproportionate stratified sampling. The estimated sample size of 2000 was based on a dementia prevalence of 10%, derived from the WiSE 2013 study, which gave sufficient precision with a margin of error of 2% and a statistical power of 0.8, with type 1 error at 0.05. The estimated design effect after oversampling on age (those ≥75) and ethnicity (Malay and Indian) was 1.934.

### 2.2 Participants

Older adults were invited to participate in the study through a letter sent to their residential address and home visits by trained interviewers. The invitation letters were sent in two languages—English and the language spoken by the respondent's ethnic group (Chinese, Malay, or Tamil). The exclusion criteria were (1) the inability to provide informed consent by the older adult and the absence of a suitable legal representative for this purpose and (2) older adults who had moved away to another address or were living outside the country during the entire study period.

A caregiver, whom the older adult or the family identified as involved in the care or care‐related decisions, was also recruited and interviewed to provide information about the older adult and to assess the caregiving burden and quality of life. If there were multiple caregivers, the person responsible for most of the care decisions or the person rated by the older adult as the one who knew them the best was selected. Paid caregivers were excluded from the survey. In addition, for participants who were cognitively capable and functioning independently, an informant (the person who knew the older adult best) was interviewed to ensure that the history provided by the older respondent was accurate. Inclusion criteria for the caregiver/informant were—being at least 21 years of age (age of majority in Singapore), able and willing to provide information on the older adult, and able to speak and understand English, Chinese (or dialects, i.e., Hokkien, Teochew and Cantonese), Malay, or Tamil.

### 2.3 Questionnaires

The study involved extensive assessment of both the respondent and the informant, with several questionnaires that assessed multiple domains such as mental health, cognition, functioning, and medical history, as well as utilization of services by the older adult, which were part of the 10/66 protocol.^6^

Briefly, 10/66 dementia diagnosis was established using:

- The *Geriatric Mental State* (*GMS*) assessment. The GMS is a semi‐structured mental state interview that applies a computer algorithm, the Automated Geriatric Examination of Computer Assisted Taxonomy (AGECAT), identifying organicity (probable dementia), psychosis, depression, and anxiety across five levels of psychopathology from 0 (non‐case) to 5 (severe case).^13^
- The *Community Screening Instrument for Dementia* (*CSI'D*),^14^ which incorporates the Consortium to Establish a Registry for Alzheimer's Dementia (CERAD) animal naming verbal fluency task, and the modified CERAD 10‐word list learning task with delayed recall,^15^, ^16^ which generates the global cognitive score (COGSCORE), an item‐weighted total score of the participants. The *CSI'D* (*Informant*)^14^ was administered to informants to establish the informants’ observations of cognitive and functional decline in the respondent. It generates the informant score known as the Relationship Score (RELSCORE), an unweighted total score from the interview.

Other assessments included the Neurological Examination (*NEUROEX)* ‐ a brief structured physical and neurological examination of the older adult, and the *Health Status Questionnaire*, which included a chronic conditions checklist and the World Health Organization Disability Assessment Schedule 2.0 (WHO‐DAS 2.0).^17^ The severity of dementia in all participants was assessed using a computerized operationalization of the Clinical Dementia Rating (CDR) scale.^18^ Interviewers also obtained information on the age, ethnicity, educational level, and marital and employment status of the older adult, and a list of the household assets using a structured questionnaire. The treatment gap of dementia was assessed by asking the respondent and the informant if they/the older adult “had ever been diagnosed with dementia or memory problems by a doctor.”

The survey was offered in English, Chinese (including dialects), Malay, or Tamil. Interviews were conducted face‐to‐face by trained interviewers through computer‐assisted personal interviews (CAPIs). Each interview took about 2 to 2.5 h.

### 2.4 Interviewer training and quality control

An established commercial survey firm conducted the survey. All the interviewers recruited to conduct the study were 21 years of age or older and had adequate experience in fieldwork. They attended a 2‐week extensive training that involved lectures, videos, and hands‐on training (on approaching the households, administering neurocognitive tests, mental state assessments, consent taking, survey administration, etc.). All interviewers were evaluated individually by trained and experienced study team members, and those who did not pass were not allowed to do the fieldwork. Five batches of interviewer training were conducted. The research team implemented stringent quality control (QC) measures, including field observations of the initial interviews by the research team to ensure that the interviewers captured the data accurately and appropriately, and monitoring the progress of the survey and visitation records to detect any discrepancies for subsequent clarification and resolution. A selection of 20% of the cases per interviewer for QC was done through phone calls or house visits. Concerns and problems that surfaced during the QC were discussed with the principal investigator and acted upon immediately. The data were stored securely on a server that was accessible only to authorized personnel, and the data set was transferred to the research team with adherence to data safety protocols. Data safety protocols included transferring data through a secure network with access limited to only the data manager from the project team and the data team from the commercial survey firm. Data sets were password protected, and personal identifiers were excluded to mitigate the risks of data linkage. A trained statistician ensured the quality of the data by conducting interim analysis.

### 2.5 Ethics

The Institutional Research and Review Committee of the Institute of Mental Health, the National Healthcare Group Domain Specific Review Board, and the SingHealth Centralised Institutional Review Board approved the study protocol. Written informed consent was obtained from all participants or their legally acceptable representatives as applicable in the form of a signature or thumbprint.

### 2.6 Statistical analyses and 10/66 dementia diagnosis

We conducted data analyses using weighted data that were adjusted for oversampling and non‐response, and post‐stratified to align with the age and ethnicity distributions of Singapore's older adult resident population. Analyses were performed using a complex survey data procedure implemented in Stata version 17.0. The 10/66 dementia diagnosis was defined as those scoring above a cut point of predicted probability of DSM‐IV dementia syndrome from the logistic regression equation developed in the 10/66 international pilot study, using coefficients from the GMS, CSI'D informant, cognitive test interviews, and the modified CERAD 10‐word list learning tasks.^19^ Descriptive analyses were performed to establish the prevalence and treatment gap of dementia and to describe the sociodemographic profile of the study population. Sociodemographic and clinical correlates of 10/66 dementia were examined using multivariable logistic regression. Significance differences in the prevalence rates between the two survey data sets (WiSE‐2023 vs. WiSE‐2013) were tested using a chi‐square test. A multivariate decomposition model for the nonlinear response model was used to determine factors associated with the change in the prevalence of dementia between WiSE 2013 and WiSE 2023, which decomposed the source of differences into two components to identify whether the overall change in the prevalence of dementia was due to different population characteristics (population or characteristics changes) or different relationships between the characteristics and dementia (coefficient changes). This model has been used previously to identify factors for the trend change.^20^, ^21^, ^22^ Statistical significance was set at the conventional level of *p* < 0.05.

## 3 RESULTS

### 3.1 Prevalence of dementia and dementia severity

A total of 2010 respondents completed the WiSE 2023 study and constituted a representative sample of the resident population 60 years of age or older living in Singapore. The response rate among eligible older adults was 62.7%, slightly lower than that observed in the earlier WiSE 2013 study of 65.6%. The response rates by ethnic groups in the WiSE 2023 survey were 50.8%, 70.9%, and 71.8% among the Chinese, Malay, and Indian ethnic groups, whereas in the WiSE 2013 study, they were 54.6%, 77.7%, and 74% among the Chinese, Malay, and Indian ethnic groups, respectively. The main reasons for refusals in both studies were similar and included the inability to commit the time for the survey due to conflicting demands and lack of interest in the study. In addition, during the WiSE 2023 survey, older adults expressed concerns about providing information to strangers, given the high incidence of scams targeted at this age group, and refused to participate in the study. Fewer than five older adults were reported to be living alone with paid caregivers in the community or in public nursing homes where the older adult was unable to provide consent due to severe physical or mental illness, and the gatekeepers stated that they did not have the contact details for the organizational caregiver. Only 1798 informants completed the survey, and all results are based on data from the 1798 dyads. The results were weighted to ensure that the prevalence data are representative of the total older population of those 60 years and above. The mean age of the respondents was 70.8 years (range, 60 to 100 years). Table 1 shows the sociodemographic distribution of the entire sample. The ethnic group that was classified as “Others” included only 17 respondents belonging to diverse and heterogeneous ethnicities. Hence, we could not make any meaningful inferences and will not discuss this group separately in this article.

**TABLE 1:** Sociodemographic distribution of the sample in 2023 and 2013.

	2023			2013		
	Un‐weighted	Un‐weighted	Weighted	Un‐weighted	Un‐weighted	Weighted
Sociodemographic characteristics	*n* (*n *= 2010)	%	%	*n* (*n *= 2565)	%	%
Age group (years)						
60–74	1090	54.2	75.0	1494	58.3	75.0
75–84	580	28.9	18.7	669	26.1	19.5
85+	340	16.9	6.3	402	15.7	5.5
Gender						
Men	908	45.2	47.1	1117	43.5	44.1
Women	1102	54.8	52.9	1448	56.5	55.9
Ethnicity						
Chinese	678	33.7	81.6	1012	39.5	83.3
Malay	699	34.8	10.4	745	29.0	9.3
Indian	616	30.6	6.4	772	30.1	6.0
Others	17	0.8	1.5	36	1.4	1.4
Marital status						
Never married	114	5.6	9.1	136	5.3	8.0
Married/cohabiting	1168	58.2	66.2	1484	57.9	64.0
Widowed	617	30.8	18.7	836	32.6	22.5
Divorced/separated	107	5.3	6.0	107	4.2	5.5
Education status						
None	217	10.8	7.2	511	20.0	16.5
Some, but did not complete primary	391	19.5	20.6	620	24.3	23.9
Completed primary	568	28.4	26.5	640	25.1	24.8
Completed secondary	545	27.2	28.2	517	20.3	22.4
Completed tertiary	282	14.1	17.6	262	10.3	12.4
Employment status						
Paid work (part‐time and full‐time)	633	31.6	42.2	688	27.1	33.9
Unemployed	35	1.7	1.3	32	1.3	1.5
Homemaker	515	25.7	16.8	808	31.9	26.3
Retired	821	41.0	39.7	1006	39.7	38.3
Received any income, benefits, or allowances monthly						
No	135	6.8	6.3	372	14.5	10.6
Yes	1864	93.2	93.7	2188	85.5	89.4
Presence of any comorbid physical disorder^a^						
No	540	26.9	35.6	719	28.0	31.5
Yes	1470	73.1	64.4	1846	72.0	68.5
Participants with informant						
No	212	10.5	13.2	144	5.6	7.7
Yes	1798	89.5	86.6	2421	94.4	92.3

^a^ Includes hypertension, heart trouble (heart attack, angina, heart failure, valve disease, and others), stroke, diabetes, and transient ischemic attacks (TIAs).

The WiSE 2023 found that the weighted prevalence of dementia using the 10/66 criteria was 8.8% (95% confidence interval [CI]: 7.3–10.7) in the older adult population. Translating this figure into Singapore's population in 2022 would mean that 73,918 older adults had dementia. When the prevalence rate of dementia in WiSE 2023 was compared to that of 10% (95% CI: 8.7–11.5) in WiSE 2013, a non‐significant decrease of 1.2% percentage points, or 12% (95% CI: −1.1 to 3.5), was observed (*p*‐value = 0.298) (Table 2).

**TABLE 2:** Comparison of prevalence of dementia in 2013 versus 2023.

	WiSE 2013			WiSE 2023				
Dementia 10/66	Unweighted (*N*)	Projected number	Weighted %	Unweighted (*N*)	Projected number	Weighted %	*χ* ^2^	*p*‐value
No	2022	465,435	90.0	1482	764,882	91.2	1.1	0.298
Yes	399	51,934	10.0	316	73,918	8.8		

### 3.2 Decomposition analysis of the change in dementia prevalence

Multivariate nonlinear decomposition analysis showed that ≈84.7% of the overall decrease in the prevalence of dementia was associated with differences in the population's composition/characteristics, and the remaining 15.3% was due to differences in the covariate effect (Table 3). The decrement in the proportion of “homemakers” of 9.3% and the increment in the proportion of “paid work” of 8.2% in the sampled population across the two surveys revealed a significant positive 24.1% and 42.3% association with the decline in the prevalence of dementia. An increase in the proportion of older adults with secondary and tertiary education had a statistically significant effect on the change in dementia prevalence. The compositional changes of older adults with secondary and tertiary education from 2013 to 2023 of 5.7% and 5.2%, respectively, showed a significant 4.5% and 10.6% positive association with the decline in dementia prevalence. The decline in stroke prevalence of 1.2% was associated with a positive 4.9% decrease in dementia prevalence.

**TABLE 3:** Multivariate nonlinear composition analysis of change in dementia between 2013 and 2023.

Category	Coef. (E)	*p*‐value	95% CI lower	95% CI upper	Coef. percentage^**a**^	Coef. (C)	*p*‐value	95% CI lower	95% CI upper	Coef. percentage^a^
Total					84.7					15.3
Age					−3.8					9.6
60–74	−0.00011	0.000	−0.00015	−0.00006	−0.8	0.00155	0.846	−0.01416	0.01726	11.0
75–84	0.00000	0.976	−0.00006	0.00006	0.0	−0.00010	0.867	−0.00131	0.00110	−0.7
85+	−0.00043	0.000	−0.00059	−0.00027	−3.1	−0.00009	0.847	−0.00106	0.00087	−0.7
Gender					0.2					0.7
Male	0.00002	0.931	−0.00037	0.00040	0.1	−0.00070	0.857	−0.00833	0.00693	−5.0
Female	0.00002	0.931	−0.00037	0.00040	0.1	0.00080	0.857	−0.00790	0.00951	5.7
Ethnicity					−0.1					−11.7
Chinese	0.00000	0.921	−0.00008	0.00009	0.0	−0.00176	0.860	−0.02141	0.01789	−12.5
Malay	−0.00003	0.357	−0.00010	0.00003	−0.2	−0.00006	0.847	−0.00063	0.00052	−0.4
Indian	0.00001	0.352	−0.00002	0.00004	0.1	0.00017	0.855	−0.00164	0.00198	1.2
Marital					0.4					25.3
Never married	−0.00023	0.370	−0.00074	0.00027	−1.6	0.00018	0.849	−0.00166	0.00201	1.3
Married	0.00006	0.707	−0.00027	0.00039	0.5	0.00313	0.855	−0.03033	0.03658	22.1
Widowed	0.00013	0.711	−0.00057	0.00083	0.9	0.00099	0.853	−0.00949	0.01147	7.0
Divorced/separated	0.00009	0.459	−0.00015	0.00034	0.7	−0.00072	0.850	−0.00822	0.00677	−5.1
Education					28.4					−2.8
None	0.00195	0.012	0.00043	0.00346	13.8	−0.00003	0.895	−0.00050	0.00044	−0.2
Some did not complete primary	0.00023	0.410	−0.00032	0.00079	1.7	0.00033	0.855	−0.00325	0.00392	2.4
Completed primary	−0.00030	0.103	−0.00065	0.00006	−2.1	−0.00047	0.855	−0.00556	0.00461	−3.4
Completed secondary	0.00063	0.195	−0.00032	0.00159	4.5	−0.00085	0.849	−0.00962	0.00792	−6.0
Completed tertiary	0.00150	0.072	−0.00014	0.00314	10.6	0.00063	0.850	−0.00587	0.00713	4.5
Employment					61.0					4.7
Employed	0.00597	0.000	0.00417	0.00778	42.3	0.00199	0.849	−0.01857	0.02255	14.1
Homemaker	0.00340	0.000	0.00179	0.00501	24.1	−0.00037	0.851	−0.00428	0.00353	−2.7
Retired	−0.00075	0.000	−0.00100	−0.00051	−5.3	−0.00095	0.850	−0.01087	0.00896	−6.8
Presence of Chronic Physical Conditions										
Hypertension	−0.00078	0.058	−0.00159	0.00003	−5.5	0.00144	0.854	−0.01386	0.01673	10.2
Diabetes	0.00001	0.455	−0.00002	0.00004	0.1	0.00037	0.862	−0.00382	0.00456	2.6
Heart problem	−0.00001	0.933	−0.00018	0.00017	−0.1	0.00011	0.879	−0.00130	0.00152	0.8
Depression	0.00007	0.084	−0.00001	0.00014	0.5	0.00019	0.861	−0.00189	0.00226	1.3
Stroke	0.00069	0.000	0.00031	0.00107	4.9	−0.00007	0.880	−0.00092	0.00079	−0.5
TIA	−0.00019	0.629	−0.00094	0.00057	−1.3	−0.00001	0.961	−0.00051	0.00049	−0.1

Abbreviation: C, Coefficient due to differences in the covariate effect; E, Coefficient due to differences in the population's composition/characteristics; TIA, transient ischemic attack.

^a^ The effects for categorical variables were normalized to ensure that the sum of the detailed coefficients effects attributed to the dummy variables is not invariant to the choice of the reference category or the omitted category.^22^

### 3.3 Severity of dementia

**FIGURE 1:**
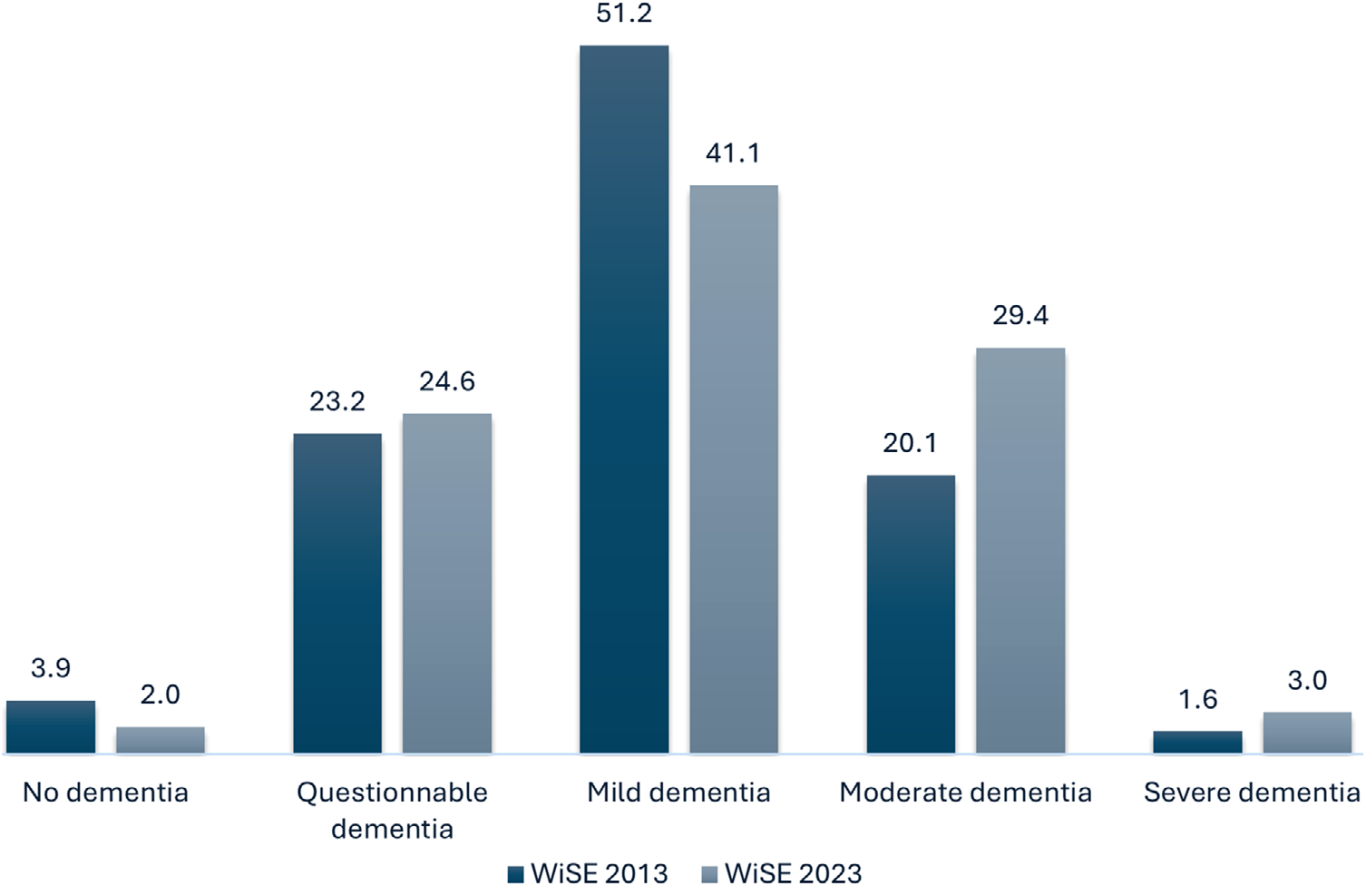
Prevalence of dementia by severity.

Using the CDR severity rating, 41.1% of those with 10/66 dementia were classified as mild, 29.4% as moderate, and 3% as severe cases. Of the cases with 10/66 dementia, 2% and 24.5% were assessed as no dementia and questionable dementia, respectively, by the CDR. Comparisons of dementia severity with the WiSE 2013 study^5^ found significant changes in the distribution, with the proportion of no dementia (2.0% vs 3.9%) and mild cases (41.1% vs 51.2%) being lower, and moderate (29.4% vs 20.1%) and severe cases (3.0% vs 1.6%) being higher in the current study (Figure 1).

### 3.4 Sociodemographic and clinical correlates of dementia

The prevalence of dementia among different sociodemographic groups is shown in Table S1. A multivariable logistic regression analysis was used to determine the significant dementia‐related factors. The significant factors associated with dementia were older age, i.e., 75 years and older as compared to ages 60–74 years (75–84 years, and 85 years and over; odds ratio (OR): 3.1, 95% CI: 1.6–6.2, and OR: 11.2, 95% CI: 5.0–24.9, respectively), Indian ethnicity as compared to Chinese ethnicity (OR: 1.7, 95% CI: 1.1–1.7), homemakers (OR: 9.4, 95% CI: 2.1–41.7), and those who had retired (OR: 11.3, 95% CI: 2.4–53.0) as compared to those who were employed. Individuals who reported being diagnosed with a stroke by a doctor (OR: 6.5, 95% CI: 2.5–16.6) or experiencing episodes of depression in their lifetime (OR: 2.8, 95% CI:1.2–6.1) had higher odds of developing dementia compared to those without a history of stroke or depression, respectively. Gender and doctor‐diagnosed hypertension, heart trouble, DM, and transient ischemic attacks were not associated with dementia (Table 4).

**TABLE 4:** Socio‐demographic and clinical factors associated with dementia.

Variables	Odds ratio^a^	95% CI	*p*‐value
Age group, y			
60–74	1 (reference)		
75–84	3.1	1.6‐6.2	0.001
85+	11.2	5.0‐24.9	<0.001
Gender			
Male	1 (reference)		
Female	1.5	0.7‐3.5	0.297
Ethnicity			
Chinese	1 (reference)		
Malay	1.5	0.9‐2.5	0.165
Indian	1.7	1.1‐2.9	0.046
Others	5.1	1.4‐18.3	0.012
Marital			
Never married	1.2	0.3‐5.0	0.822
Married	1 (reference)		
Widowed	1.4	0.7‐2.8	0.324
Divorced/separated	0.1	0.‐0.3	0.002
Education			
Completed tertiary	1 (reference)		
None	2.7	0.9‐8.2	0.090
Some did not complete primary	2.3	0.8‐6.7	0.118
Completed primary	1.7	0.6‐4.9	0.307
Completed secondary	0.7	0.2‐2.0	0.486
Employment			
Employed^b^	1 (reference)		
Unemployed	3.6	0.3‐44.0	0.322
Homemaker	9.4	2.1‐41.7	0.003
Retired	11.3	2.4‐53.0	0.002
Hypertension			
No	1 (reference)		
Yes	0.9	0.5‐1.7	0.834
Heart problems			
No	1 (reference)		
Yes	1.1	0.6‐2.2	0.672
Diabetes			
No	1 (reference)		
Yes	1.0	0.6‐1.8	0.986
TIAs			
No	1 (reference)		
Yes	1.6	0.4‐6.4	0.485
Stroke			
No	1 (reference)		
Yes	6.5	2.5‐16.6	<0.001
Depression^c^			
No	1 (reference)		
Yes	2.8	1.2‐6.1	0.013

Abbreviation: TIA, transient ischemic attack.

^a^ Odds ratio was derived from multiple logistic regressions using the enter method.

^b^ Employed—were employed either full time or part time.

^c^ Lifetime depression based on the respondent/informant self‐report.

### 3.5 Treatment gap of dementia

The weighted proportion of those who met the criteria for dementia but had not been diagnosed by a doctor for memory problems or dementia at the point of the survey (i.e., undiagnosed dementia) was 51.5% (*n* = 152). Among those diagnosed by the doctor, 34.2% (*n* = 37) had received a prescription for a medication specifically for dementia (e.g., cholinesterase inhibitor or *N*‐methyl‐d‐aspartate [NMDA] receptor blocker).

A significant decrease was observed in the prevalence of undiagnosed dementia across the two surveys. Comparing the prevalence of undiagnosed dementia in WiSE 2023 to that observed in WiSE 2013, we found that the prevalence of undiagnosed dementia had decreased significantly by 19.1%, from 70.6% to 51.5% (*p*‐value = 0.002), which translates into a percentage decrease of 27.1% in the treatment gap of dementia.

## 4 DISCUSSION

### 4.1 Dementia prevalence

Using a nationally representative sample and the same methodology across studies, we found that the prevalence of dementia decreased by 1.2 percentage points from 10% to 8.8% over 10 years in Singapore. Although the difference was not statistically significant, it is in line with studies done elsewhere. Using data derived from a cohort of 21,442 older adults, the Health and Retirement Study (HRS) conducted in the United States showed that the age‐adjusted prevalence of dementia exhibited a notable decrease within the 65 years and older demographic from 12.2% in 2000 (95% CI, 11.7%–12.7%) to 8.5% in 2016 (7.9% to 9.1%)—a statistically significant decline of 3.7 percentage points.^23^ A systematic review of 43 articles undertaken in 2018, to understand the global changes in dementia prevalence and incidence over time yielded diverse findings.^24^ Examination of the prevalence rates disclosed an upward trend in record‐based surveys and cohort studies conducted in Japan, Canada, and France. Conversely, prevalence rates remained stable, primarily from cohort studies in Sweden, Spain, and China. In contrast, more recent studies (since 2010) reported a significant decline in prevalence, particularly in European regions (e.g., the United Kingdom and Sweden) and the United States.^24^ Using data from the Global Burden of Disease (GBD) Study, Avan and Hachinski^25^ analyzed trends in the absolute number and age‐standardized rates of incidence of dementia. The results showed that although the absolute number of new cases of dementia is increasing globally due to population aging, 71 countries showed declining trends in the age‐standardized rate of dementia incidence from 1990 to 2019. Our study methodology did not allow us to examine the incidence of dementia in Singapore. Regardless, the decreasing incidence identified by Avan and Hachinski^25^ strengthens the case for prevention strategies against dementia risk factors, which could potentially lead to a global decline in the incidence and even the prevalence of dementia.

### 4.2 Factors associated with dementia

A noticeable rise in dementia prevalence was observed with advancing age, particularly among those 85 years of age and over—a finding consistently echoed in various studies within the existing body of literature. It has been suggested that a higher prevalence of cardiometabolic diseases may increase the risk of dementia in this age group.^26^ Reduction in physical activity and depression and delirium have been identified as additional factors elevating dementia risk in this demographic.^27^ Age‐related brain changes, such as atrophy in the hippocampus, an imbalance of amyloid beta (Aβ) production and degradation, and activation of inflammation, have also been implicated.^28^, ^29^

Individuals of Indian ethnicity were significantly more likely to have dementia compared to those of Chinese ethnicity. We found no significant differences in mean age among the three ethnic groups. In terms of educational status, more individuals of Indian ethnicity held tertiary qualifications than those of Chinese and Malay ethnic groups. However, they had a higher prevalence of doctor‐diagnosed stroke and self‐reported depression compared to those of Chinese ethnicity. Both stroke and depression may increase the risk of dementia (discussed in greater detail below). Other studies have also noted ethnic differences. A recent study in the United Kingdom examined anonymized electronic primary care records for adults 65 years of age and older from 1997 to 2018.^30^ The study revealed higher rates of hypertension, dyslipidemia, obesity, and diabetes in minority ethnic groups compared to White individuals. The researchers concluded that these common risk factors pose a greater risk of dementia in people of South Asian origin, particularly cardiovascular risk factors such as abnormal lipid profiles, hypertension, diabetes, and obesity. They also suggested that risk factor severity may be elevated in minority ethnic groups, either due to a specific risk factor (e.g., higher blood glucose levels in those with diabetes) or prolonged exposure to risk factors.^30^

Significantly higher rates of dementia were found among individuals who were retired and full‐time homemakers compared to their counterparts who were employed. This being a cross‐sectional study, causality is difficult to establish. Although employment may confer a certain degree of protection against cognitive decline by promoting physical and mental activity and social connectedness,^31^, ^32^ it has also been suggested that retirement may be a trigger for cognitive decline.^33^, ^34^ Finally, it is also possible that individuals with cognitive decline choose to retire from their jobs due to their inability to cope with the job demands.^35^

The association between dementia and stroke observed in the current study is well‐established in the literature. The two chronic illnesses are closely associated, whether in the form of vascular cognitive impairment or Alzheimer's disease. A systematic review that included 36 studies of prevalent stroke (1.9 million participants) and 12 studies of incident stroke (1.3 million participants) found that for prevalent stroke, the pooled hazard ratio for all‐cause dementia was 1.69 (95% CI: 1.49–1.92; *p* < 0.00001). For incident stroke, the pooled risk ratio was 2.18 (95% CI: 1.90–2.50; *p* < 0.00001).^36^ In addition to the damage caused to the neuronal tissue, stroke may trigger a neurodegenerative process by disrupting amyloid clearance or activating autoimmune responses to post‐stroke brain antigens.^37^

A lifetime history of depression was also associated with a higher prevalence of dementia. Several hypotheses have been proposed to explain this association, including depression as an independent risk factor for developing dementia; dementia or cognitive impairment as features of depression; depression serving as a prodrome of dementia; depression as a reaction to cognitive decline; and shared common risk factors such as cardiovascular disease between dementia and depression.^38^, ^39^ Regardless of the cause, our findings underscore the importance of treating individuals with depression and screening those with late‐life depression for dementia.

### 4.3 Increase in the diagnosis of dementia and treatment

Our study found that about 50% of individuals with dementia were diagnosed by a doctor as having dementia/memory problems—a sharp contrast to the previous study, where only ≈30% of those assessed as having dementia during the survey were diagnosed by a doctor as having dementia. Comparing the prevalence of undiagnosed dementia in WiSE 2023 to that observed in WiSE 2013, we found that the prevalence of undiagnosed dementia had decreased significantly by 19%, from 70.6% to 51.5%. A systematic review of 23 studies indicated that the pooled rate of undetected dementia was 61.7% (95% CI: 55.0%–68.0%). Higher rates of under‐detection were observed in China and India compared to Europe and North America, in community settings (vs residential/nursing care), among individuals younger than 70 years of age, in males, and when diagnosed by a general practitioner.^40^ Furthermore, about 34% of individuals with doctor‐diagnosed dementia in Singapore were receiving medications specifically for the management of dementia. This improvement reflects the multiple efforts in Singapore to improve awareness and early diagnosis of dementia.

### 4.4 Strengths and limitations of the study

The study's strengths include its large sample size, the inclusion of a representative sample of the general population that included individuals who could speak only local dialects, single‐phase assessment using validated assessments and questionnaires, and superior QC processes. However, certain limitations need to be acknowledged. First, our response rate was 62.7%, and it is possible that individuals who refused to participate were more physically or mentally disabled. The prevalence of dementia and depression could have been higher in this group. However, most of the older adults who refused to participate in the study told the interviewers that they were concerned aboutthe survey being a scam, the length of the survey, or were wary of the risk of COVID‐19 infection. However, in cases where household members acted as gatekeepers and refused access to older adults, it was not possible to assess the reasons for refusal. Because the survey has a cross‐sectional design, we are unable to establish any temporal relationships between dementia, stroke, and depression. In addition, we could also not perform any hearing tests, although hearing loss has been significantly associated with cognitive decline. The diagnosis of dementia by a doctor was based on self‐report, and we were not able to verify it with administrative data because personal identifiers were not linked to data to ensure participant confidentiality. Finally, older adults and their caregivers may have been reluctant to talk about low mood as well as risk factors such as financial difficulties and loneliness due to social desirability bias.

## 5 CONCLUSIONS

Although there is a decrease in the prevalence of dementia, we acknowledge that the lack of a significant change could mean that there is no change in the prevalence of dementia. Therefore, active aging and dementia awareness initiatives, as well as strengthening primary care to ensure early diagnosis and treatment of those with dementia, must continue to be well funded. These programs must be evaluated to ensure that they continue to impact Singaporeans positively and that the gains observed are maintained. Our study has identified several modifiable risk factors, which include stroke and depression. Early diagnosis and treatment of both of these conditions can prevent the risk of cognitive decline and functional impairment. Dementia awareness initiatives in Singapore must go beyond symptom identification and highlight the risk factors and preventive strategies. Future research should focus on a deeper understanding of ethnic factors influencing dementia risk. The prevalence and severity of comorbid medical conditions, lifestyle, and other psychosocial risk factors must be explored across ethnic groups. The current study focused on dementia among adults 60 years of age or older; future epidemiological studies must examine early‐onset dementia, which is emerging as a growing concern globally.

## CONFLICT OF INTEREST STATEMENT

The authors declare no conflicts of interest.

## CONSENT STATEMENT

All the participants or their legally approved representatives provided written informed consent.

## Supporting information



Supporting Information

Supporting Information
