# Supplementary material for: Prevalence of dementia in Singapore: Changes across a decade
Source: Alzheimers Dement. 2025 Jan 27;21(2):e14485. doi: 10.1002/alz.14485 (PMC11848337; doi:10.1002/alz.14485)
Supplement: Supplementary file 1 — Supporting Information [file ALZ-21-e14485-s002.docx]

| Variables | | Unweighted N | Weighted % | 95% CI |
| --- | --- | --- | --- | --- |
| Age group** | 60-74 | 34 | 3.0 | (1.9,4.7) |
|  | 75-84 | 110 | 18.2 | (13.2,24.5) |
|  | 85+ | 172 | 48.6 | (38.8,58.6) |
| Gender * | Men | 114 | 6.3 | (4.5,8.7) |
|  | Women | 202 | 11.1 | (8.8,13.9) |
| Ethnicity | Chinese | 66 | 8.7 | (6.9,10.9) |
|  | Malay | 131 | 8.9 | (7.2,10.9) |
|  | Indian | 111 | 9.6 | (7.5,12.3) |
|  | Others | 8 | 10.8 | (6.3,17.8) |
| Education** | None | 95 | 30.8 | (22.0,41.2) |
|  | Some, but did not complete Primary | 89 | 14.9 | (10.6,20.5) |
|  | Completed primary | 63 | 6.6 | (4.1,10.5) |
|  | Completed secondary | 46 | 3.1 | (1.8,5.3) |
|  | Completed Tertiary | 20 | 3.6 | (1.6,7.7) |
| Marital status** | Never married | 11 | 5.9 | (2.3,14.4) |
|  | Married/ Cohabiting | 112 | 5.7 | (4.2,7.8) |
|  | Widowed | 186 | 23.6 | (18.4,29.7) |
|  | Divorced | 7 | 1.1 | (0.4,2.8) |
| Employment** | Employed | 9 | 0.5 | (0.1,2.2) |
|  | Unemployed | 1 | 1.9 | (0.3,13.1) |
|  | Homemaker | 143 | 14.5 | (10.4,19.8) |
|  | Retired | 161 | 15.0 | (11.9,18.9) |

**Supplementary Table 1 Prevalence (95% CI) of dementia by demographic and socioeconomic characteristics**

Chi Square test = *: p value < 0.01; **: p value < 0.001
